# Identifying Frailty and Its Associated Factors Among Patients With Traumatic Brain Injury in Vietnam

**DOI:** 10.1002/brb3.71885

**Published:** 2026-11-03

**Authors:** Tung Hoang Tran, Hai Minh Vu, Tham Thi Nguyen, Carl Latkin, Laurent Boyer, Bastien Boussat, Pascal Auquier, Huyen Do, Cyrus S. H. Ho

**Affiliations:** ^1^ Faculty of Medicine VNU University of Medicine and Pharmacy, Vietnam National University Hanoi Vietnam; ^2^ Institute of Orthopaedics and Trauma Surgery Vietnam–Germany Friendship Hospital Hanoi Vietnam; ^3^ Department of Trauma Thai Binh University of Medicine and Pharmacy Hung Yen Vietnam; ^4^ CEReSS‐Health Service Research and Quality of Life Center, Assistance Publique‐Hôpitaux de Marseille Aix‐Marseille University Marseille France; ^5^ Bloomberg School of Public Health Johns Hopkins University Baltimore Maryland USA; ^6^ Department of Clinical Epidemiology Grenoble Alpes University Grenoble France; ^7^ Department of Health Policy and Management, College of Health Science Korea University Seoul Republic of Korea; ^8^ Institute of Health Economics and Technology (iHEAT) Hanoi Vietnam; ^9^ Department of Psychological Medicine, Yong Loo Lin School of Medicine National University of Singapore Singapore Singapore; ^10^ Faculty of Public Health VNU University of Medicine and Pharmacy, Vietnam National University Hanoi Vietnam

**Keywords:** frailty, post‐discharge care, rehabilitation, traumatic brain injury (TBI)

## Abstract

**Background:**

Traumatic brain injury (TBI) is a leading cause of long‐term disability and mortality, particularly in low‐ and middle‐income countries (LMICs), where post‐discharge rehabilitation services are often limited. Frailty is a multidimensional construct encompassing physical, cognitive, psychological, and social vulnerability. After TBI, multidimensional frailty may overlap with disability, depressive symptoms, persistent neurological problems, and social vulnerability. However, little is known about Groningen Frailty Indicator (GFI)‐defined frailty among adults after hospital discharge for TBI in resource‐limited settings.

**Methods:**

A cross‐sectional study was conducted among 212 adult patients discharged after TBI from Thai Binh General Hospital, Vietnam. Data were collected via structured telephone interviews during a study period extending from January to August 2020. Frailty was measured using the GFI and operationally defined as a score of ≥ 4. Multivariable logistic regression was used to examine factors associated with GFI‐defined frailty status.

**Results:**

The prevalence of GFI‐defined frailty was 36.8%, despite the majority (93.9%) having mild TBI. In the adjusted logistic model, female sex was associated with lower odds of GFI‐defined frailty than male sex (aOR = 0.37; 95% CI: 0.15–0.87), and rural residence was associated with lower odds than urban residence (aOR = 0.24; 95% CI: 0.09–0.66). Current smoking (aOR = 2.80; 95% CI: 1.10–7.14), post‐injury sequelae (aOR = 2.39; 95% CI: 1.15–4.98), depressive symptoms (aOR = 3.24; 95% CI: 1.23–8.54), and friend‐group support (aOR = 1.58; 95% CI: 1.07–2.34) were associated with higher odds of GFI‐defined frailty. Overall, 33.0% of participants reported post‐injury sequelae, and 10.2% experienced catastrophic health expenditure. Friend‐group support was positively associated with frailty, suggesting that higher reported support from friends may reflect greater support needs among frail patients after discharge rather than a harmful effect of social support.

**Conclusions:**

GFI‐defined frailty was common among reachable adults discharged after TBI from this single hospital. The identified relationships are cross‐sectional associations and may partly reflect overlap between frailty, functional limitation, psychological burden, and social‐support needs. Post‐discharge follow‐up should consider functional, psychological, social, occupational, and financial vulnerability in addition to neurological symptoms. Multicenter longitudinal studies incorporating pre‐injury status and rehabilitation exposure are needed to confirm these findings.

## Graphical abstract

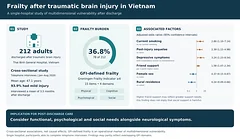

## 1 Background

Traumatic brain injury (TBI) is a leading cause of death and long‐term disability worldwide, affecting individuals across all age groups, with a disproportionate impact on those of working age (Bennett et al. 2016; Greenspan et al. 1996). A global systematic review estimated that approximately 69 million people experience TBI each year, with low‐ and middle‐income countries (LMICs) bearing a disproportionate share of the burden (Dewan et al. 2019). In these settings, suboptimal road infrastructure, limited enforcement of safety regulations, and under‐resourced trauma and rehabilitation systems contribute to the high burden of TBI Mass et al. 2022. Common causes include road traffic injuries, falls, and occupational injuries. Beyond physical impairments such as paralysis, motor dysfunction, and speech disorders, TBI often leads to significant psychological sequelae, including depression, anxiety, and sleep disturbances, which collectively reduce quality of life and increase long‐term health system burdens (Viola‐Saltzman & Watson et al. 2012; Zgaljardic et al. 2015). While multidisciplinary rehabilitation and structured follow‐up services have expanded in many high‐income settings, access to comprehensive post‐discharge assessment and long‐term rehabilitation remains limited or fragmented in many LMICs (Mass et al. 2022).

Frailty is a multidimensional construct reflecting reduced reserve and increased vulnerability across physical, cognitive, psychological, and social domains (Xue 2011). As articulated by Rockwood and Mitnitski (2007), frailty reflects the cumulative accumulation of health deficits over time (Rockwood & Mitnitski, 2007). Although extensively studied in geriatric populations, frailty is increasingly recognized as a critical issue among individuals with acute or chronic injuries, including those with TBI. Most existing TBI studies have assessed pre‐injury frailty in older adults as a prognostic marker for mortality, functional outcome, length of stay, or discharge destination (Cray et al. 2025; Galimberti et al. 2022). Considerably less is known about multidimensional frailty measured after hospital discharge, particularly among adults across a broader age range.

The application of frailty measures after TBI also presents an important conceptual challenge. Frailty after TBI is difficult to distinguish from overlapping post‐injury problems, including disability, depressive symptoms, persistent post‐concussive symptoms, sleep disturbance, and social vulnerability (Boucham et al. 2024; Manley et al. 2025; McIntosh et al. 2025). This distinction is particularly important when multidimensional instruments such as the Groningen Frailty Indicator (GFI) are used, because they may capture physical, psychological, and social consequences of injury as part of the frailty construct (Peters et al. 2012). Moreover, the GFI was developed and validated primarily in older populations, which warrants caution when applying its threshold to adults across a broad age range. In the present study, GFI‐defined frailty was therefore interpreted as a multidimensional marker of post‐discharge vulnerability rather than as a distinct biological syndrome caused by TBI.

Vietnam, a lower middle‐income country experiencing rapid urbanization and motorization, exemplifies this challenge. In 2025, national statistics recorded 18,371 road traffic crashes, resulting in 10,337 deaths and 12,197 injuries (General Statistics Office of Vietnam, 2026). These figures indicate a continuing burden of road trauma, an important cause of TBI, although they do not directly quantify the incidence of TBI or the number of survivors with long‐term neurological consequences. Despite this burden, few studies have systematically investigated post‐discharge GFI‐defined frailty and its associated factors among adults with TBI in Vietnam or comparable LMIC settings. This limited evidence constrains understanding of the functional, psychological, and social needs that may persist after hospital discharge. Against this backdrop, the present study was undertaken to (1) estimate the prevalence of GFI‐defined frailty, and (2) examine factors associated with GFI‐defined frailty status among adults discharged after TBI from a single hospital in Vietnam. The study was intended to provide hospital‐based evidence to inform context‐appropriate post‐discharge assessment and future rehabilitation research in resource‐limited settings.

## 2 Methods

### 2.1 Study Setting and Participants

This cross‐sectional study was conducted at the Traumatology Department, Thai Binh General Hospital, Vietnam, between January and August 2020. A total of 216 patients were initially recruited based on predefined eligibility criteria. After data screening, four participants were excluded because of substantial missing information on key study variables. The final analytic sample included 212 participants, accounting for 98.1% of the recruited patients.

In this study, TBI was operationally defined as a hospital diagnosis of traumatic intracranial injury coded as S06 according to the International Classification of Diseases, 10th Revision (ICD‐10). The diagnosis was confirmed through a review of medical records and discharge documentation. Clinically, TBI was diagnosed by attending physicians based on a documented history of head trauma, neurological assessment, and cranial computed tomography findings when clinically indicated. ICD‐10 code S06 includes concussion, traumatic cerebral edema, diffuse or focal brain injury, epidural hemorrhage, subdural hemorrhage, traumatic subarachnoid hemorrhage, and other or unspecified intracranial injuries (World Health Organization, 2019).

Inclusion criteria comprised (1) a medically documented TBI diagnosis based on ICD‐10 code S06 and completion of hospital treatment, defined as having received a physician's order for hospital discharge; (2) age of 18 years or older; and (3) current residence in Vietnam. Because data were collected through telephone interviews and several instruments relied on self‐reported information, participants were required to have sufficient cognitive and communication ability to understand and respond to the questionnaire. Patients with severe cognitive impairment or inability to communicate effectively with trained data collectors were therefore excluded.

When necessary, caregivers assisted participants by clarifying factual information, such as household circumstances and healthcare use. However, subjective measures, including depressive symptoms, sleep quality, perceived social support, and frailty, were obtained directly from participants whenever reliable self‐report was possible. Patients whose cognitive or communication impairment prevented reliable participation, even with caregiver assistance, were excluded. The potential selection bias introduced by this exclusion criterion is acknowledged in the limitations paragraph in Section 4.

### 2.2 Data Collection and Measurements

Data were collected through telephone interviews, each lasting approximately 20 min. This method was chosen for its cost‐effectiveness, efficiency, and feasibility in maintaining post‐discharge contact with participants. Written informed consent was obtained from eligible participants during hospitalization, before discharge. At the beginning of each telephone interview, trained data collectors reintroduced the study purpose, confirmed the participant's identity, and obtained verbal confirmation of continued willingness to participate before administering the questionnaire. Participants were informed that they could decline to answer any question or withdraw from the interview at any time.

Data collectors received standardized training to ensure consistency and quality in administering the questionnaire by phone. A pilot study was conducted with 10 individuals of diverse ages, sex, and occupational backgrounds to pretest and refine the instrument. The final semi‐structured questionnaire comprised seven main sections.

#### 2.2.1 Socioeconomic Status

Participants were asked to report demographic and socioeconomic characteristics, including sex (male/female), occupation (freelance worker, white‐collar worker, blue‐collar worker/farmer, or student/unemployed), educational attainment (primary/junior high school, high school, intermediate/ college/ vocational, tertiary or upper, or uneducated), marital status (single, married/partnered, or divorced/separated/widowed), place of residence (urban/rural), average monthly household income (in USD), and comorbidities (categorized as 0, 1, or ≥ 2 co‐existing chronic conditions). Caregiver information was collected and categorized into three groups: parents/spouse, offspring, and grandchildren/helpers. These caregiver categories were not mutually exclusive, as participants could report more than one type of caregiver.

#### 2.2.2 Health Risk Behaviors

Tobacco and alcohol use were assessed to characterize participants’ health‐related risk behaviors. Smoking status was determined by self‐reported cigarette use within the past 30 days.

Alcohol consumption was evaluated using the Alcohol Use Disorders Identification Test‐Consumption (AUDIT‐C), a validated three‐item screening tool assessing drinking frequency, quantity, and frequency of heavy drinking episodes (six or more drinks) (Bush et al. 1998). The total score ranges from 0 to 12, with thresholds of ≥ 4 for males and ≥ 3 for females indicating hazardous drinking.

#### 2.2.3 Perceived Social Support

Social support was assessed using the Multidimensional Scale of Perceived Social Support (MSPSS) (Kieu,^2023^). This instrument measures perceived support from three distinct sources: family, friends, and significant others. Each domain is rated on a 7‐point Likert scale, with higher scores indicating stronger perceived support from the corresponding source.

#### 2.2.4 Clinical Characteristics

Information on TBI was obtained from medical records and participant interviews and included the cause of injury, severity (mild/moderate/severe), presence of concurrent injuries, receipt of first aid, occurrence of post‐injury sequelae, length of hospital stay, and time from discharge to interview.

TBI severity was classified according to the Glasgow Coma Scale (GCS) score documented in the medical record. Mild TBI was defined as GCS 13–15, moderate TBI as GCS 9–12, and severe TBI as GCS 3–8. This classification reflects the commonly used clinical grading of TBI severity based on GCS; however, GCS alone may not fully capture the heterogeneity of TBI, including neuroimaging findings, post‐traumatic amnesia, duration of loss of consciousness, and other clinical features (Manley et al. 2025). Accordingly, the GCS categories used in this study should be interpreted as a pragmatic clinical classification rather than a comprehensive characterization of TBI severity.

Injury severity was further assessed using the Injury Severity Score (ISS), which aggregates injuries across six anatomical regions: head/neck, face, chest, abdomen, extremities, and external (Manley et al. 2025). Scores range from 0 to 75, with a score of 75 assigned in cases involving injuries classified as untreatable (AIS score of 6).

The interval between hospital discharge and the telephone interview was calculated using the documented discharge date and the interview date.

#### 2.2.5 Catastrophic Health Expenditure

To estimate catastrophic health expenditure due to TBI, we calculated total direct medical costs by summing expenditures on medicines, diagnostic tests, medical examinations and hospital fees, hospital bed charges, and other related costs.

Household capacity to pay was defined as (monthly income − monthly spending) × 12. Catastrophic health expenditure was defined as TBI‐related healthcare expenditure equal to or greater than 40% of the household's estimated annual capacity to pay.

#### 2.2.6 Mental Health and Sleep Quality

Depressive symptoms were assessed using the Patient Health Questionnaire‐9 (PHQ‐9). This scale consists of nine items scored from 0 (*not at all*) to 3 (*nearly every day*), yielding a total score from 0 to 27 (Kroenke et al. 2001). Severity levels were categorized as minimal (0–4), mild (5–9), moderate (10–14), moderately severe (15–19), and severe depressive symptoms (20–27).

Sleep quality was measured using the Pittsburgh Sleep Quality Index (PSQI), which assesses seven domains of sleep disturbances (Buysse et al. 1989). Each component is scored from 0 to 3, with total scores ranging from 0 to 21. A total score ≥ 5 indicates poor sleep quality, whereas scores < 5 represent good sleep quality.

#### 2.2.7 Frailty Assessment

Frailty was evaluated using the GFI, a 15‐item questionnaire covering physical, cognitive, psychological, and social domains. Total scores range from 0 to 15, with higher scores indicating greater multidimensional vulnerability. Participants were classified as having “GFI‐defined frailty” if they had a GFI score of ≥ 4, and as not having GFI‐defined frailty if they had a GFI score of < 4 (Peters et al. 2012; Steverink, 2001).

Because the GFI includes psychological and social as well as physical and cognitive domains, GFI‐defined frailty in this study was interpreted as a multidimensional marker of post‐discharge vulnerability. It was not assumed to represent a distinct biological syndrome caused by TBI. This interpretation is particularly relevant because some GFI domains may overlap conceptually with depressive symptoms, post‐injury sequelae, sleep problems, and perceived social support.

### 2.3 Statistical Analysis

All statistical analyses were performed using Stata version 16 (StataCorp LLC, College Station, TX, USA). Missing data were assessed before analysis. Four participants with substantial missing information, including missing data on the frailty outcome, were excluded; therefore, descriptive analyses were performed using available data for each variable. Multivariable logistic regression was conducted using complete‐case analysis, with the analytical sample restricted to participants with complete data on the outcome and all variables included in the model. No imputation was performed.

Categorical variables were summarized as frequencies and percentages and compared between frailty groups using chi‐squared tests. The distribution of continuous variables was assessed using skewness and kurtosis tests for normality. Normally distributed continuous variables were compared between groups using independent‐samples *t*‐tests, whereas non‐normally distributed continuous variables were compared using the Mann–Whitney *U* test. Continuous variables were presented as mean ± standard deviation, or the median and interquartile range (Q1–Q3) for non‐normally distributed data.

Frailty status was analyzed as a dichotomous outcome using the operational cut‐off of GFI ≥ 4. Binary logistic regression was used to examine factors associated with GFI‐defined frailty. Results are presented as adjusted odds ratios (aORs) with 95% confidence intervals (CIs). Because frailty was relatively common in this sample, odds ratios from the logistic model were interpreted strictly as odds ratios and not as prevalence ratios or risk ratios.

Candidate variables were selected based on clinical relevance and previous literature. These included sociodemographic characteristics, socioeconomic status, comorbidities, residence, injury‐related factors, time from discharge to interview, depressive symptoms, sleep quality, and perceived social support.

A forward stepwise selection strategy was applied with an entry threshold of *p* < 0.20 to reduce the number of candidate variables given the modest sample size. This stepwise procedure was used only as a pragmatic variable‐reduction approach for exploratory modelling, rather than as a causal variable‐selection strategy or as a procedure for developing a definitive prediction model.

Potential collinearity was considered during model specification by avoiding simultaneous retention of variables that measured highly overlapping constructs. No formal collinearity threshold was used as a post‐selection decision rule. Associations involving depressive symptoms, sleep quality, post‐injury sequelae, and perceived social support were interpreted cautiously because these variables may overlap conceptually with domains included in the GFI.

Interaction terms were not included because no interaction effects were specified a priori and the number of frailty events was limited. Statistical significance was defined as a two‐sided *p*‐value of less than 0.05.

The conceptual framework and data analysis plan were performed using the BETAMED software (https://nghiencuu.online/), created and maintained by iHEAT.vn.

## 3 Results

**Table** **1** shows that a total of 212 individuals with a history of TBI were included in the analysis. Of these, 67.5% were male, 42.9% reported working as freelance laborers, and 84.9% resided in rural areas. Overall, 78 of 212 participants (36.8%) met the criterion for GFI‐defined frailty. The majority were married or partnered (70.3%) and received caregiving support primarily from parents or spouses (73.1%). Among participants with available expenditure data, 10.2% experienced catastrophic health expenditure. In terms of health‐related risk behaviors, 23.1% reported hazardous alcohol use, while 76.9% were current smokers. The mean age of participants was 47.1 years (SD = 17.6). Compared with non‐frail participants, those with frailty were more likely to live in urban areas (24.4% vs. 9.7%), whereas rural residence was more common among non‐frail participants than among frail participants (90.3% vs. 75.6%; *p* < 0.01). Current smoking was also significantly more common in the frailty group than in the non‐frailty group (84.6% vs. 72.4%; *p* = 0.04).

**TABLE 1:** Demographic characteristics of participants (n = 212).

Characteristics	No frailty (n = 134)	Frailty (n = 78)	Total (N = 212)	*p*‐value
Sex, n (%)				> 0.05
Male	84 (62.7)	59 (75.6)	143 (67.5)	
Female	50 (37.3)	19 (24.4)	69 (32.5)	
Occupation, n (%)				0.27
Freelance workers	56 (41.8)	35 (44.9)	91 (42.9)	
White‐collar workers	11 (8.2)	8 (10.3)	19 (9.0)	
Blue‐collar workers/farmers	55 (41.0)	23 (29.5)	78 (36.8)	
Students/unemployed	12 (9.0)	12 (15.4)	24 (11.3)	
Educational, n (%)				0.96
Primary/junior high school	65 (48.5)	36 (46.2)	101 (47.6)	
High school	43 (32.1)	26 (33.3)	69 (32.5)	
Intermediate/college/vocational education	14 (10.4)	8 (10.3)	22 (10.4)	
Tertiary or upper	9 (6.7)	7 (9.0)	16 (7.5)	
Uneducated	3 (2.2)	1 (1.3)	4 (1.9)	
Current location, n (%)				< 0.01
Urban	13 (9.7)	19 (24.4)	32 (15.1)	
Rural	121 (90.3)	59 (75.6)	180 (84.9)	
Marital status, n (%)				0.40
Single	29 (21.6)	20 (25.6)	49 (23.1)	
Married/partnered	98 (73.1)	51 (65.4)	149 (70.3)	
Divorced/separated/widowed	7 (5.2)	7 (9.0)	14 (6.6)	
Hazardous drinking, n (%)	34 (25.4)	15 (19.2)	49 (23.1)	0.31
Current smoking, n (%)	97 (72.4)	66 (84.6)	163 (76.9)	0.04
Number of comorbidities, n (%)				0.26
0	102 (76.1)	55 (70.5)	157 (74.1)	
1	26 (19.4)	15 (19.2)	41 (19.3)	
≥ 2	6 (4.5)	8 (10.3)	14 (6.6)	
Caregiver, n (%)				
Parents/spouse	103 (76.9)	52 (66.7)	155 (73.1)	0.11
Offspring	42 (31.3)	32 (41.0)	74 (34.9)	0.15
Grandchildren/helpers	12 (9.0)	8 (10.3)	20 (9.4)	0.75
Catastrophic health expenditure, n (%)^a^	12 (9.1)	9 (12.2)	21 (10.2)	0.48
Age, year^b^				0.09
Mean (SD)	45.4 (16.4)	49.8 (19.2)	47.1 (17.6)	
Median (Q1‐Q3)	46.5 (31.0–57.0)	52.0 (33.0–66.0)	48.0 (32.0–60.5)	
Monthly income, USD^b^				0.25
Mean (SD)	474.7 (213.6)	460.3 (251.5)	469.4 (227.8)	
Median (Q1–Q3)	431.0 (301.7–603.4)	431.0 (258.6–560.3)	431.0 (301.7–603.4)	

^a^ Available data: n = 206 (6 missing); all other variables had complete data.

^b^ Continuous variables were compared using the Mann–Whitney *U* test due to non‐normal distributions.

**Table** **2** presents the clinical characteristics and levels of social support among the participants. The leading cause of TBI was traffic accidents, accounting for 139 cases (65.6%). The vast majority of patients (93.9%) sustained mild TBI, while 33.0% experienced post‐injury sequelae. TBI combined with other injuries was observed in 61.3% of participants, including 64.2% of the non‐frail group and 56.4% of the frailty group. Furthermore, 78.3% of participants reported minimal or no depressive symptoms, and none had severe symptoms. Compared with non‐frail participants, those with frailty had higher proportions of moderate/severe TBI (11.5% vs. 3.0%; *p* = 0.01), post‐injury sequelae (44.9% vs. 26.1%; *p* < 0.01), and mild to moderately severe depressive symptoms (33.3% vs. 14.9%; *p* < 0.01). The mean Injury Severity Score (ISS) was 9.8 (SD = 6.5). The ISS was also significantly higher in the frailty group than in the non‐frailty group (11.8 vs. 8.6; *p* < 0.01). The average duration of hospitalization was 11.6 days (SD = 9.3), and the mean time from discharge to interview was 3.5 months (SD = 1.8). Regarding social support, mean scores for the family, friend, and significant others support domains were 5.4 (SD = 0.8), 4.7 (SD = 0.9), and 5.0 (SD = 1.0), respectively. No significant differences were observed between the frailty and non‐frailty groups across the three social support domains. The mean score on the GFI was 2.8 (SD = 1.9), with substantially higher scores observed among frail participants than among non‐frail participants (5.0 vs. 1.4; *p* < 0.01).

**TABLE 2:** Clinical and social support of participants (n = 212).

Characteristics	No frailty (n = 134)	Frailty (n = 78)	Total (N = 212)	*p*‐value
Cause of TBI, n (%)				0.03
Falls	23 (17.2)	20 (25.6)	43 (20.3)	
Traffic accidents	86 (64.2)	53 (67.9)	139 (65.6)	
Other causes	25 (18.7)	5 (6.4)	30 (14.2)	
TBI severity, n (%)				0.01
Mild	130 (97.0)	69 (88.5)	199 (93.9)	
Moderate/severe	4 (3.0)	9 (11.5)	13 (6.1)	
First aid, n (%)	93 (69.4)	45 (57.7)	138 (65.1)	0.08
TBI combined with other injuries, n (%)^a^	86 (64.2)	44 (56.4)	130 (61.3)	0.25
Sequelae, n (%)^a^	35 (26.1)	35 (44.9)	70 (33.0)	< 0.01
Depression, n (%)				< 0.01
Minimal or none	114 (85.1)	52 (66.7)	166 (78.3)	
Mild/moderate/moderately severe	20 (14.9)	26 (33.3)	46 (21.7)	
Sleep quality, n (%)				0.21
Good	100 (74.6)	52 (66.7)	152 (71.7)	
Poor	34 (25.4)	26 (33.3)	60 (28.3)	
ISS score (0–75)^a^ ^b^				< 0.01
Mean (SD)	8.6 (5.8)	11.8 (7.1)	9.8 (6.5)	
Median (Q1–Q3)	6.0 (3.0–11.0)	11.0 (6.0–18.0)	9.0 (6.0–14.0)	
Time in hospital, days^b^				0.08
Mean (SD)	11.1 (9.6)	12.5 (8.7)	11.6 (9.3)	
Median (Q1–Q3)	10.0 (6.0–13.0)	11.5 (8.0–16.0)	11.0 (7.0–14.0)	
Time from discharge to interview, months^b^				0.81
Mean (SD)	3.5 (1.8)	3.6 (1.8)	3.5 (1.8)	
Median (Q1–Q3)	3.0 (2.0–5.0)	4.0 (2.0–5.0)	3.0 (2.0–5.0)	
Social support				
Family support (1–7)^c^				0.75
Mean (SD)	5.4 (0.9)	5.4 (0.8)	5.4 (0.8)	
Friend support (1–7)^c^				0.37
Mean (SD)	4.7 (1.0)	4.8 (0.9)	4.7 (0.9)	
Significant‐other support (1–7)^c^				0.48
Mean (SD)	5.0 (1.1)	5.2 (0.9)	5.0 (1.0)	
Median (Q1–Q3)	5.0 (4.8–5.8)	5.0 (4.5–6.0)	5.0 (4.8–6.0)	
GFI score (0–15)^b^				< 0.01
Mean (SD)	1.4 (0.7)	5.0 (1.0)	2.8 (1.9)	
Median (Q1–Q3)	1.0 (1.0–2.0)	5.0 (4.0–6.0)	2.0 (1.0–4.0)	

Abbreviations: GFI, Groningen Frailty Indicator; ISS, injury severity score; TBI, traumatic brain injury.

^a^ Available data were n = 209, n = 191, and n = 210 for TBI combined with other injuries, sequelae, and ISS score, respectively; all other variables had complete data.

^b^ Continuous variables were compared using the Mann–Whitney *U* test due to non‐normal distributions.

^c^ Continuous variables were approximately normally distributed and compared using the independent‐samples *t*‐test.

Factors associated with GFI‐defined frailty among participants are summarized in **Table** **3**. Female patients had lower odds of GFI‐defined frailty (aOR = 0.37; 95% CI: 0.15–0.87) than their male counterparts. Similarly, rural residents had lower odds of GFI‐defined frailty (aOR = 0.24; 95% CI: 0.09–0.66) than urban residents. Current smokers had higher odds of GFI‐defined frailty (aOR = 2.80; 95% CI: 1.10–7.14) than non‐smokers. Post‐injury sequelae and depressive symptoms were also positively associated with frailty. Patients with sequelae had higher odds of GFI‐defined frailty (aOR = 2.39; 95% CI: 1.15–4.98) than those without sequelae. Similarly, patients with mild, moderate, or moderately severe depressive symptoms had higher odds of GFI‐defined frailty (aOR = 3.24; 95% CI: 1.23–8.54) than those with minimal or no depressive symptoms. Each 1‐point increase in the friend support score was associated with higher odds of GFI‐defined frailty (aOR = 1.58; 95% CI: 1.07–2.34).

**TABLE 3:** Multivariable logistic regression analysis identifying factors associated with frailty among participants.

Factor	Frailty status		
	aOR	95% CI	*p*‐value
Socio‐economic characteristics			
Sex (vs. male)			
Female	0.37	0.15–0.87	0.02
Current location (vs. urban)			
Rural	0.24	0.09–0.66	0.01
Offspring caregiver (vs. no)			
Yes	2.10	0.97–4.53	0.06
Current smoking (vs. no)			
Yes	2.80	1.10–7.14	0.03
Clinical			
First aid (vs. no)			
Yes	0.58	0.28–1.20	0.14
Cause of TBI (vs. fall)			
Traffic accidents	0.90	0.37–2.17	0.81
Others	0.30	0.07–1.22	0.09
Sequelae (vs. no)			
Yes	2.39	1.15–4.98	0.02
Depression (vs. minimal or none)			
Mild/moderate/moderately severe	3.24	1.23–8.54	0.02
Social interaction			
Friend group	1.58	1.07–2.34	0.02

*Note*: GFI‐defined frailty was classified as GFI ≥ 4.

Abbreviations: CI, confidence interval; GFI, Groningen frailty indicator; OR, odds ratio; TBI, traumatic brain injury.

## 4 Discussion

This study is among the few conducted in LMICs that systematically evaluate GFI‐defined frailty as a multidimensional marker of post‐discharge vulnerability among patients following TBI. While much of the existing evidence is derived from high‐income countries with established systems for multidisciplinary rehabilitation, frailty after TBI remains under‐recognized and under‐studied in LMICs. This is particularly concerning because TBI is a leading cause of long‐term disability among adults in these settings, where access to post‐discharge care, psychosocial support, and functional follow‐up is often limited or fragmented. However, because the present study was conducted at a single hospital, its findings should be interpreted as hospital‐based evidence rather than as estimates representative of Vietnam or LMICs more broadly. Our findings showed that 36.8% of patients were classified as frail based on the GFI. Direct comparison with previous TBI studies should be made cautiously because estimates vary according to patient age, injury severity, clinical setting, timing of assessment, and the frailty instrument used (Cray et al. 2025). For instance, research conducted by Tang and colleagues reported a frailty prevalence of 18% among patients with TBI in the United States (Tang et al. 2022); however, differences in the study populations and frailty definitions limit direct comparison with our estimate. These findings indicate that a mild GCS‐based classification may coexist with persistent clinical, functional, psychological, social, or financial vulnerability after discharge (Manley, McIntosh et al. 2025). Nevertheless, because pre‐injury frailty and functional status were not measured, the study cannot determine whether these vulnerabilities developed after TBI, were present before the injury, or reflected a combination of both.

Multivariable logistic regression analysis identified several factors associated with GFI‐defined frailty, including male sex, urban residence, post‐injury sequelae, depressive symptoms, current smoking, and friend‐group support. Given the multidimensional structure of the GFI, these findings should be interpreted as associations with GFI‐defined post‐TBI vulnerability rather than as evidence that these factors independently cause frailty. Female participants had lower odds of GFI‐defined frailty than male participants, while rural residents had lower odds of GFI‐defined frailty than urban residents. These findings should not be interpreted as evidence that female sex or rural residence has a protective effect. They may reflect differences in injury characteristics, healthcare‐seeking patterns, post‐discharge circumstances, unmeasured socioeconomic conditions, or residual confounding within this hospital‐based sample. A recent systematic review found substantial sex‐related differences in symptom burden and recovery after mild TBI, with women frequently reporting greater symptom burden or slower recovery in several domains (Arachchi et al. 2026). The contrast with our findings reinforces the need for context‐specific and cautious interpretation of sex‐related associations.

Current smoking was associated with higher odds of GFI‐defined frailty. Recent longitudinal and mechanistic studies have also reported associations between current or long‐term smoking and frailty, although this evidence was derived mainly from middle‐aged and older populations without TBI (Jung et al. 2025; Kojima et al. 2018). However, because smoking and frailty were assessed during the same study period, the temporal direction of this relationship cannot be determined. Smoking may reflect broader behavioral or health vulnerability, but the present findings do not establish that smoking caused or increased the risk of frailty. Similarly, post‐injury sequelae were associated with higher odds of GFI‐defined frailty. This relationship may partly reflect overlapping manifestations of post‐injury vulnerability because persistent neurological, physical, or cognitive problems may also be represented within the GFI (Manley et al. 2025; McIntosh et al. 2025).

Depressive symptoms were associated with higher odds of GFI‐defined frailty, consistent with previous evidence describing a close relationship between psychological burden and multidimensional frailty (Choi et al. 2022; Skogstad et al. 2014; Wiseman et al. 2013). However, this association requires particularly cautious interpretation because the GFI itself includes a psychological domain. Depressive symptoms and frailty may be bidirectionally related, may arise from shared clinical or social circumstances, or may partly measure overlapping aspects of psychological vulnerability. The association should therefore be interpreted as an observed relationship rather than evidence that depression independently causes frailty.

Friend‐group support was also associated with higher odds of GFI‐defined frailty. This finding does not indicate that support from friends is harmful. A plausible explanation is support mobilization or reverse directionality: participants with greater functional, psychological, or social needs may receive or perceive more support from friends after discharge. Perceived social support may also overlap conceptually with the social domain of the GFI. Recent longitudinal evidence indicates that the relationship between social support and frailty may vary according to the type of support, frailty definition, and transition being examined, supporting cautious interpretation of cross‐sectional associations (Barghouth et al. 2024).

One strength of the study was the use of established instruments, including the GFI, PHQ‐9, PSQI, and MSPSS, which enabled assessment across physical, psychological, cognitive, and social dimensions. Data collection at an average of 3.5 months after discharge also allowed the study to characterize vulnerability during an early post‐discharge period, although participants were not all interviewed at the same interval and individual recovery trajectories could not be assessed.

Several limitations should be acknowledged. First, the cross‐sectional design precludes causal inference and does not establish the temporal sequence between frailty, depressive symptoms, smoking, sequelae, and perceived social support. Second, the relatively small sample and single‐site design limit generalizability, particularly because 93.9% of participants had mild TBI according to their documented GCS scores. The findings may therefore not apply to other hospitals, geographical areas, or populations with greater proportions of moderate or severe TBI. Third, telephone interviews may be affected by recall error and social desirability bias, particularly for psychological symptoms and health‐related behaviors. Although caregivers could assist in clarifying factual information, when necessary, subjective measures depended primarily on participant self‐report, and the extent of caregiver assistance may have varied. Fourth, excluding patients with cognitive or communication impairment severe enough to prevent reliable participation may have introduced selection bias. Patients who could not be contacted or participate reliably, even with caregiver assistance, may have represented a more vulnerable subgroup; consequently, the prevalence of GFI‐defined frailty may have been underestimated. Fifth, the timing of interviews varied among participants, and recovery status may have differed according to the interval since discharge.

Residual confounding and measurement overlap should also be considered. The study lacked detailed information on pre‐injury function, baseline frailty, cognitive impairment severity, psychiatric history, socioeconomic deprivation beyond income, and rehabilitation exposure. In particular, the receipt, timing, intensity, and continuity of physical therapy, occupational therapy, neurorehabilitation, outpatient follow‐up, mental health referral, and return‐to‐work support were not measured adequately. These factors may have been associated with both post‐TBI recovery and GFI‐defined frailty. Moreover, depressive symptoms, sleep quality, post‐injury sequelae, and perceived social support may partly capture psychological, physical, cognitive, or social vulnerability already represented within the GFI. These variables should therefore be interpreted as correlates of multidimensional post‐TBI vulnerability rather than independent causal predictors. The GFI was also developed primarily for use in older populations, whereas the present sample included adults across a broad age range; consequently, its established threshold should be viewed as an operational screening classification in this population. Finally, forward stepwise selection may produce unstable variable retention and confidence intervals in a modest sample, and the limited number of frailty events may reduce the precision of estimates for predictors with sparse categories (Dyer, 2025). Because frailty was relatively common, the logistic regression estimates should be interpreted as odds ratios rather than prevalence ratios or risk ratios. Future studies should consider larger samples, prespecified reduced models, penalized regression, internal validation, and direct estimation of prevalence ratios.

Despite these limitations, the study contributes valuable empirical evidence from an LMIC context. These findings support the need for a basic post‐discharge care package for TBI survivors in resource‐limited settings. The findings suggest that post‐discharge follow‐up should not be restricted to neurological symptoms alone (Nelson et al. 2025). Frailty assessment and multidisciplinary care approaches may help identify patients with broader vulnerabilities and facilitate targeted supportive management after trauma (Owodunni et al. 2026). Where feasible, a brief multidimensional assessment could include functional status, ADL/IADL dependence, persistent neurological symptoms, depressive symptoms, sleep disturbance, return‐to‐work status, caregiver availability, social‐support needs, financial burden, and access to rehabilitation or outpatient follow‐up services (Owodunni et al. 2025). Patients with multiple vulnerabilities could be considered for closer clinical follow‐up or referral to available physical rehabilitation, occupational rehabilitation, neurorehabilitation, mental health, social assistance, financial counselling, or return‐to‐work services. These recommendations should be viewed as practical considerations arising from the observed pattern of vulnerability rather than as evidence that formal frailty screening or any specific intervention improves outcomes. Multicenter longitudinal studies incorporating pre‐injury status, repeated frailty assessment, and detailed rehabilitation exposure are needed to confirm the observed associations and clarify how vulnerability changes during recovery.

## 5 Conclusion

Among 212 adults interviewed after discharge for TBI from a single hospital in Vietnam, 36.8% met the operational threshold for GFI‐defined frailty. Although most participants were classified as having mild TBI according to their documented GCS scores, the observed prevalence indicates that multidimensional vulnerability may remain present after hospital discharge.

Current smoking, post‐injury sequelae, depressive symptoms, and friend‐group support were associated with higher odds of GFI‐defined frailty, whereas female sex and rural residence were associated with lower odds of GFI‐defined frailty. The positive association between friend‐group support and frailty may reflect greater support needs among frail patients after discharge rather than a harmful effect of support from friends. Because of the cross‐sectional design, these findings represent associations and do not establish temporal or causal relationships.

In this study, GFI‐defined frailty should be interpreted as a multidimensional marker of post‐discharge vulnerability that may encompass functional limitation, psychological burden, social vulnerability, and unmet care needs, rather than as a distinct biological syndrome caused by TBI. Post‐discharge follow‐up should therefore consider functional recovery, persistent neurological symptoms, depressive symptoms, sleep problems, return‐to‐work status, caregiver and social‐support needs, financial burden, and access to rehabilitation in addition to routine neurological assessment. Multicenter longitudinal studies incorporating pre‐injury status, repeated frailty assessment, and detailed rehabilitation exposure are needed to confirm these associations and clarify how multidimensional vulnerability changes during recovery.

## Author Contributions

**Tung Hoang Tran**: conceptualization, methodology, data curation, formal analysis, investigation, writing – original draft, writing – review and editing. **Hai Minh Vu**: conceptualization, methodology, data curation, formal analysis, writing – original draft, writing – review and editing. **Tham Thi Nguyen**: data curation, investigation, writing – original draft, writing – review and editing. **Carl Latkin**: conceptualization, methodology, supervision, writing – review and editing. **Laurent Boyer**: conceptualization, writing – review and editing, supervision, methodology. **Bastien Boussat**: conceptualization, methodology, supervision, writing – review and editing. **Pascal Auquier**: conceptualization, methodology, writing – review and editing, supervision. **Huyen Do**: data curation, investigation, writing – original draft, writing – review and editing. **Cyrus S. H. Ho**: conceptualization, methodology, formal analysis, supervision, writing – review and editing, writing – original draft.

## Funding

The dataset and the original research project were supported by the Key Research Group on Injury Treatment and Prevention (ITP‐TBU) at Thai Binh University of Medicine and Pharmacy, led by A/Prof. Vu Minh Hai and Dr. Tran Hoang Tung. The present manuscript represents a secondary and advanced analysis of data generated through the original project. This research was funded by the research project QG (QG.25.188) of Vietnam National University, Hanoi. The grant provided support for research activities, investigator effort, data management and analysis, manuscript preparation, and article processing charges (APC) associated with publication.

## Ethics Statement

This study was approved by the Institutional Review Board of Thai Binh University of Medicine and Pharmacy (Decision No. 1183/HDDD, dated December 31, 2019). The research was conducted in accordance with the principles of the Declaration of Helsinki. Prior to participation, all individuals were fully informed about the study objectives, procedures, potential risks and benefits, and their rights, including the option to decline or withdraw at any stage without any negative consequences. Written informed consent was obtained from all participants. Participation was entirely voluntary. All data were anonymized, stored securely, and used exclusively for research purposes.

## Conflicts of Interest

The authors declare no conflicts of interest.

## Data Availability

The dataset used and/or analyzed during the current study is available from the corresponding author on reasonable request.
